# Nitrogen Production by Efficiently Removing Oxygen From Air Using a Perovskite Hollow-Fiber Membrane With Porous Catalytic Layer

**DOI:** 10.3389/fchem.2018.00329

**Published:** 2018-08-06

**Authors:** Tianmiao Hu, Hangyue Zhou, Hui Peng, Heqing Jiang

**Affiliations:** ^1^Qingdao Key Laboratory of Functional Membrane Material and Membrane Technology, Qingdao Institute of Bioenergy and Bioprocess Technology, Chinese Academy of Sciences, Qingdao, China; ^2^University of Chinese Academy of Sciences, Beijing, China

**Keywords:** nitrogen production, perovskite, oxygen transport membrane, hollow-fiber, catalytic layer

## Abstract

Nowadays, nitrogen is mainly produced from air by cryogenic separation, pressure-swing adsorption (PSA) and polymeric membrane technology. In this paper, we report a perovskite membrane-based nitrogen production route, which is basically driven by methane combustion. By coupling air separation with methane combustion on the opposite sides of oxygen-permeable perovskite membrane, most of oxygen in air is efficiently removed through the perovskite membrane and then consumed by methane oxidation. A nitrogen production rate of ca. 23 cm^3^ min^−1^ with purity of 98–99% was successfully achieved, and remained stable over 120 h, with a methane conversion of 71–73% on the other side of perovskite membrane. This work demonstrates that the joint use of oxygen-permeable perovskite membrane and methane oxidation is a promising strategy for nitrogen production and inspires more research efforts in the field of gas separation.

## Introduction

Nitrogen, which constitutes 78% by volume of air, is extensively applied in chemical industry, e.g., as purging gas for pipelines (Bernardo and Drioli, 2010; Ivanova and Lewis, 2012) or as feed gas in ammonia synthesis (Kyriakou et al., 2017). To date, commercial-scale nitrogen production mainly adopts cryogenic separation, pressure-swing adsorption (PSA) or polymeric membrane technology (Koros and Mahajan, 2000; Castle, 2002). Since developed by Linde (1903), cryogenic separation technology is widely used in large-scale air separation plants and can provide nitrogen with extremely ultra-high purity (99.9999%) (Ivanova and Lewis, 2012). Considering the large site area, huge capital and energy costs of developing cryogenic plants, PSA or polymeric membrane separation technology has caught worldwide interests on satisfying the demands of those air separation plants with small to medium nitrogen production capacity or without requiring ultra-high purity (Puranik et al., 2016). In membrane separation system, nitrogen/oxygen separation is driven by the gradient in concentration or partial pressure (or both) across the membrane (Gin and Noble, 2011). Under this driving force, nitrogen and oxygen are separated by polymeric membranes based on the solution diffusion mechanism (Baker and Low, 2014), which is governed by permeability and selectivity (Chong et al., 2016). However, the difference of kinetic diameters between oxygen (3.46 Å) and nitrogen (3.64 Å) is too small, and thus it is usually difficult to obtain a very high permselectivity using the polymeric membranes (Dong et al., 2013). Furthermore, polymeric membranes for gas separation are also restricted by their poor chemical and thermal stability (Robeson, 1999; Park et al., 2010).

In past decades, dense perovskite membranes, which possess mixed oxygen ionic and electronic conductivity, attract growing attentions owing to theoretically 100% permselectivity for oxygen in air separation (Feldhoff et al., 2007). At high temperature, oxygen molecules are converted to oxygen ions over perovskite membrane surface and then diffuse through membrane driven by the oxygen partial pressure gradient. As a result, oxygen can be separated from air by using perovskite type oxide membranes. Many relevant researches have been done to achieve the oxygen production *via* perovskite oxygen-permeable membranes (Shao et al., 2000; Jin et al., 2001; Luo et al., 2010), and a satisfactory oxygen purity could be achieved over 99.4% (Zhu et al., 2009; Liang et al., 2010).

In fact, the concentration of nitrogen will be significantly increased when oxygen in the stream is removed to the other side *via* perovskite oxygen-permeable membranes. Thus, it is expected that the production of nitrogen with purity of above 97% can be achieved if most of the oxygen in the fed air is efficiently removed. Our recent study has indicated the feasibility of producing nitrogen using Ce_0.9_Pr_0.1_O_2−δ_ - Pr_0.6_Sr_0.4_FeO_3−δ_ (CPO-PSFO) disk-shaped oxygen-permeable membrane. To get a higher nitrogen production rate, one can choose the membrane that shows higher oxygen permeability. In addition, the oxygen permeation flux can be further improved if the permeated oxygen can be fast consumed on the permeate side of perovskite membrane. For example, Jiang et al. (2010b) reported that, the oxygen permeation flux of a perovskite BaCo_x_Fe_y_Zr_1−x−y_O_3−δ_ (BCFZ) hollow-fiber membrane increased from 3.3 to 8 cm^3^ min^−1^ cm^−2^ when methane was fed on shell side at 875°C. Lobera et al. (2012) further observed that when dilute methane was used as sweep gas in a perovskite Ba_0.5_Sr_0.5_Co_0.8_Fe_0.2_O_3−δ_ (BSCF) membrane reactor, the logarithm of oxygen partial pressure gradient increased from 1.16 to 4.02, resulting in a 2.5-fold increase of oxygen permeation flux. Inspired by promoted oxygen removal performance, it is feasible to produce high purity nitrogen driven by an oxygen consumption reaction in perovskite membrane reactor. Compared with disk-shaped membranes, the hollow-fiber membrane, produced *via* phase inversion process, can provide higher oxygen permeation flux and larger membrane area per unit packing volume when packed into a membrane module. In this work, we combined air separation with methane combustion in a perovskite BCFZ hollow-fiber membrane reactor for nitrogen production, which has been previously used for the production of oxygen-enriched air (Hamel et al., 2006; Liang et al., 2010) and the partial oxidation or dehydrogenation of light hydrocarbons (Jiang et al., 2009, 2010a). The influences of porous catalytic layer, methane concentration and operation temperatures on nitrogen production were investigated, and the feasibility of producing nitrogen with the purity of 98–99% have been demonstrated by using perovskite BCFZ hollow-fiber membrane.

## Experimental section

### Fabrication of perovskite membranes with porous catalytic layer

Perovskite BCFZ powders were prepared through the one-pot citric acid–ethylenediaminetetraacetic acid (EDTA) complexing process, as described in detail elsewhere (Dong et al., 2010; Liang et al., 2013). The stoichiometric amounts of metal nitrates, citric acid and EDTA (the molar ratios of EDTA/citric acid/metal cations = 1:1.5:1) were dissolved in deionized water. And the pH value was adjusted to 8–9 using aqueous ammonia. After being stirred overnight, the homogenized precursor solution was heated to remove water. Then, the resultant dark gel was burnt on a hot plate and further calcined at 950°C for 5 h in muffle oven.

Dense BCFZ hollow-fiber membrane used in this work was obtained from Fraunhofer Institute for Interfacial Engineering and Biotechnology (IGB). BCFZ slurry (a mixture of BCFZ powders and deionized water) was brushed on the central part of membrane outside surface. Then the coated membrane was heated at 1050°C for 1 h to make porous layer. For ensuring isothermal condition during performance tests, BCFZ hollow-fiber membrane (except porous layer part) was coated by Au paste, and then treated at 950°C to form a dense oxygen-impermeable film.

### Characterization of materials

The crystal structure of fresh BCFZ powders was identified by X-ray diffraction (XRD, Bruker D8 Advance) equipped with a monochromator using Cu Kα radiation at room temperature. Experimental diffraction patterns were collected in a 2θ range of 20–80°. The outside surface and cross-section morphology of fresh BCFZ uncoated hollow-fiber membrane and the one with porous layer were observed by using a JEOL JSM-6700F field emission scanning electron microscope (SEM); and the morphology of hollow-fiber membrane with porous layer after measurements and 120 h operation was studied by using a Hitachi S-4800 SEM.

### Nitrogen production tests

**Figure 1:**
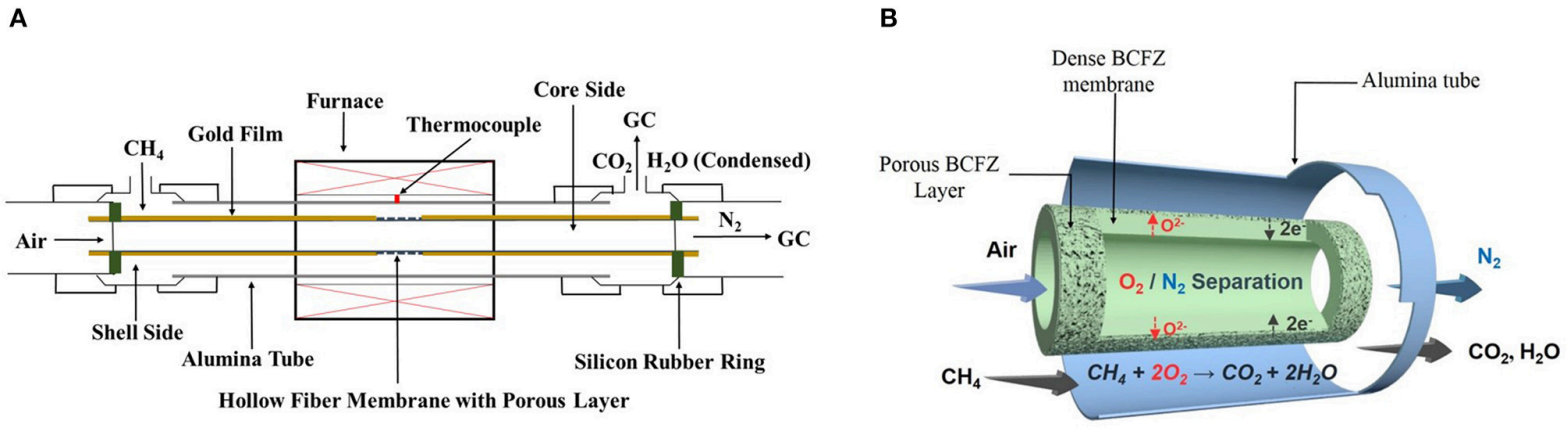
Schematic diagram of the BCFZ hollow-fiber membrane reactor **(A)** used in this work, and the scheme of nitrogen production with *in situ* removal of oxygen *via* perovskite hollow-fiber membrane **(B)**.

Nitrogen production was carried out in a lab-made high-temperature device, as shown in Figure 1A. Outside the furnace, silicon-rubber rings were used to seal the two ends of BCFZ hollow-fiber membrane. And the porous layer part was located in the middle of the furnace to make it isothermal. The sealing performance of membrane reactor was checked before nitrogen production tests. The leakages of oxygen were no more than 1%, which demonstrated a good sealing performance. Then, synthetic air was fed into core side of membrane reactor, diluted methane was fed simultaneously into shell side. The flow rates of each gas were controlled by gas mass-flow controllers. The concentrations of each gas at the exit of BCFZ hollow-fiber membrane reactor was analyzed by an on-line gas chromatograph.

The nitrogen purity (*C*_N_2__), production rate of nitrogen (*P*_N_2__), oxygen permeation flux (*J*_O_2__), methane conversion (*X*_CH_4__) and CO_2_ selectivity (*S*_CO_2__) were calculated according to following equations:

(1) $$\begin{array}{rcl} {\text{C}}_{{\text{N}}_{2}} & = & \left(1-\frac{F_{{\text{O}}_{2},\text{out}}}{F_{\text{total},\text{out}}}\right)\times 100\% \end{array}$$

(2) $$\begin{array}{rcl} {\text{P}}_{{\text{N}}_{2}} & = & F_{\text{total},\text{out}}\;\times {\text{C}}_{{\text{N}}_{2}} \end{array}$$

(3) $$\begin{array}{rcl} {\text{J}}_{{\text{O}}_{2}} & = & \frac{F_{{\text{O}}_{2},\text{in}}\;-\;F_{{\text{O}}_{2},\text{out}}}{S} \end{array}$$

(4) $$\begin{array}{rcl} {\text{X}}_{\text{C}{\text{H}}_{4}} & = & \left(1-\frac{F_{\text{C}{\text{H}}_{4},\text{out}}}{F_{\text{C}{\text{H}}_{4},\text{in}}}\right)\times 100\% \end{array}$$

(5) $$\begin{array}{rcl} {\text{S}}_{{\text{CO}}_{2}} & = & \frac{F_{\text{C}{\text{O}}_{2},\text{out}}}{F_{\text{C}{\text{H}}_{4},\text{in}}\;-\;F_{\text{C}{\text{H}}_{4},\text{out}}}\;\times 100\% \end{array}$$

Where *F*_i_ is the flow rate of gas *i, S* is the effective area of hollow-fiber membrane.

## Results and discussion

**Figure 2:**
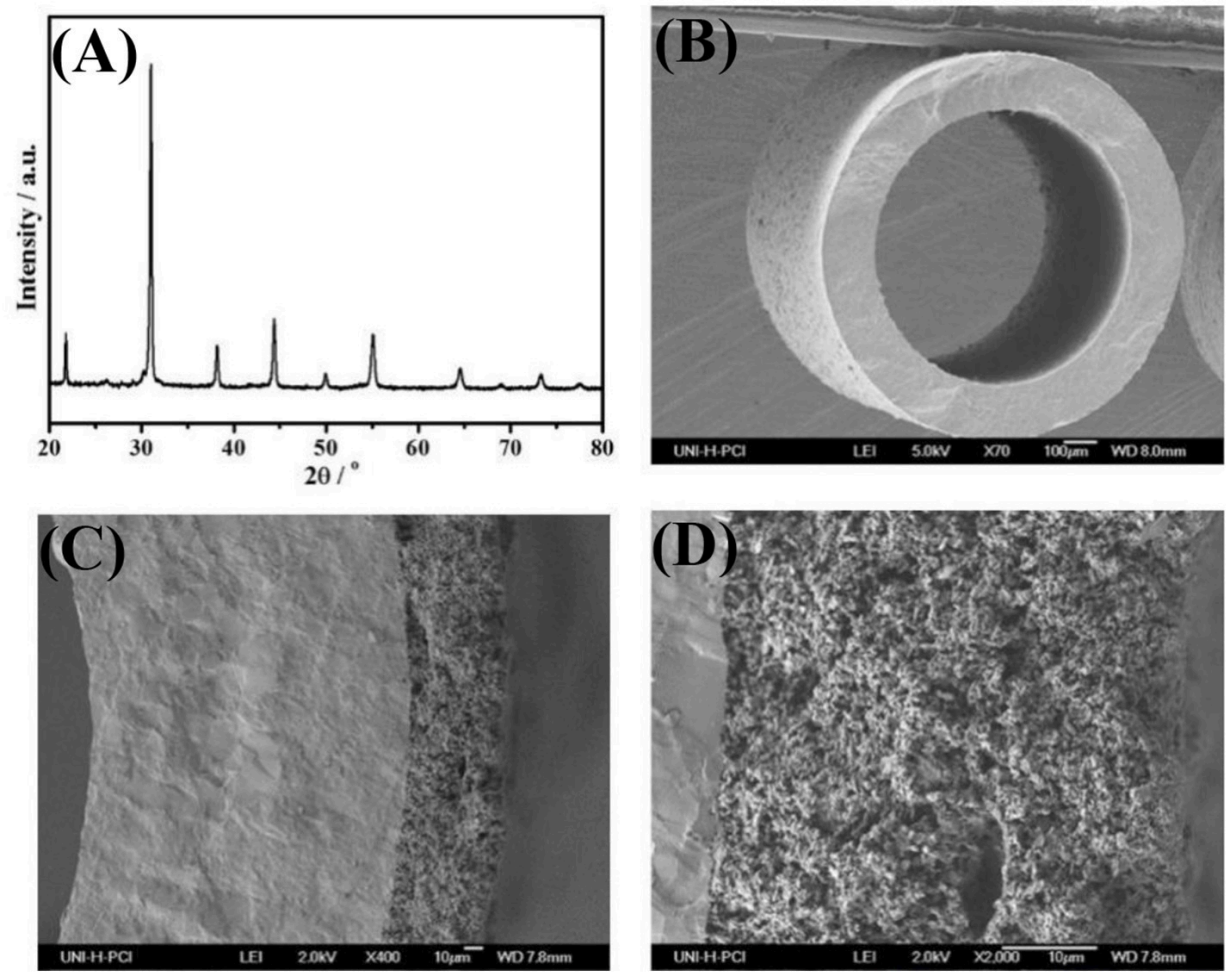
The XRD-patterns of fresh BCFZ powders **(A)** after being calcined at 950°C for 5 h, cross-section micrographs of the uncoated **(B)** and coated BCFZ hollow-fiber membrane **(C**,**D)**.

The XRD patterns of as-obtained BCFZ powder (calcined under air at 950°C for 5 h) is presented on Figure 2A. It can be found that BCFZ material possesses a typical perovskite structure, which corresponds with the observation of Wang et al. (2005). It was reported that BCFZ oxide remained its perovskite structure during the temperature increasing from 30 to 1000°C, with a heating rate of 5°C/min (Wang et al., 2005). Figures 2B–D show the cross-section micrographs of BCFZ fresh hollow-fiber with / without BCFZ porous layer. Unlike hollow-fibers with finger-like straight pores used in other works (Wang et al., 2009; Wu et al., 2013), it can be seen from Figure 2B the BCFZ hollow-fiber membrane is dense and no crack can be observed in the bulk of the membrane. This hollow-fiber membrane without finger-like pores could bring higher mechanical strength. Moreover, the outer diameter of the fiber is 800–900 μm and inner diameter is 500–600 μm. To efficiently catalyze the methane oxidation over membrane surface, in our work, a porous BCFZ layer is deposited on the outside surface of BCFZ hollow-fiber membrane followed by treating at 1050°C for 1 h. From Figure 2C, it can be seen that BCFZ porous layer is well deposited on the outside surface of dense membrane. In addition, unlike the dense membrane, the heat-treated outside BCFZ layer is still porous. The magnified image, as shown in Figure 2D, indicates that the connected BCFZ particles construct a porous three-dimensional network.

**Figure 3:**
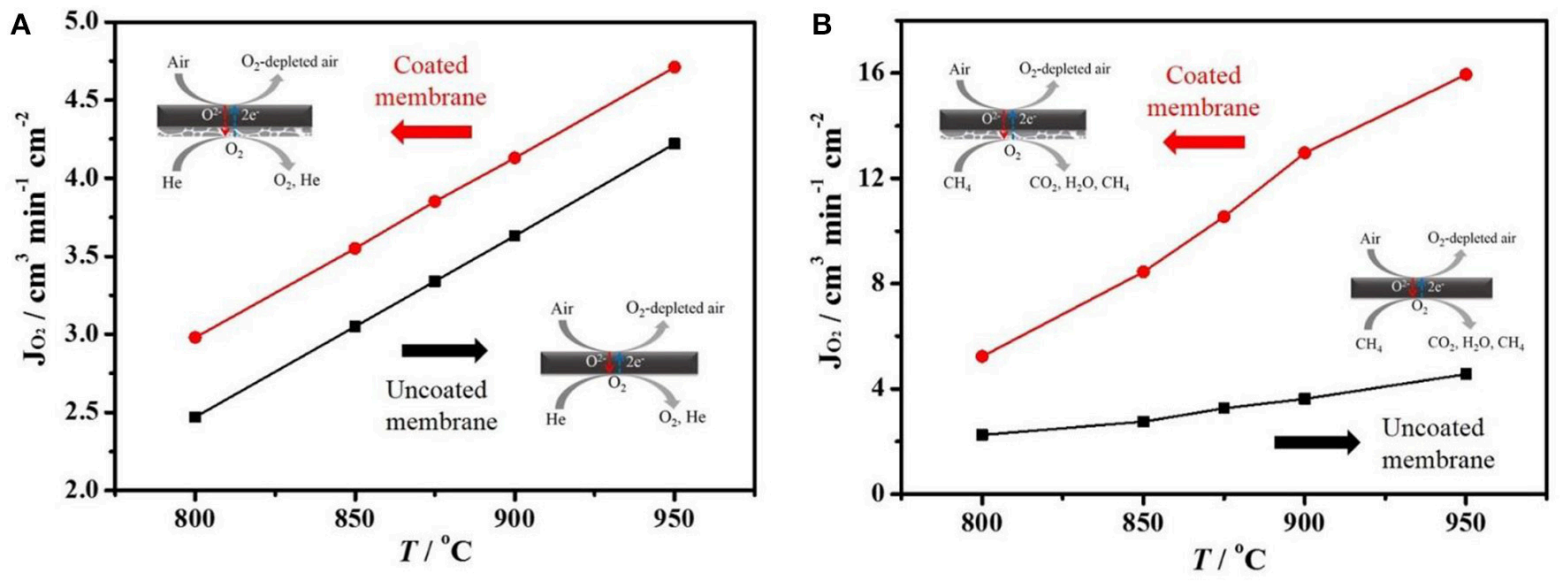
Oxygen permeation fluxes (*J*_O_2__) of a coated or uncoated BCFZ hollow-fiber membrane as a function of temperature. Membrane area: 0.8 cm^2^. **(A)** Core side: *F*_Air_ = 150 cm^3^ min^−1^. Shell side: *F*_He_ = 49 cm^3^ min^−1^, *F*_Ne_ = 1 cm^3^ min^−1^ (for calculating *J*_O_2__). **(B)** Core side: *F*_Air_ = 149 cm^3^ min^−1^, *F*_Ne_ = 1 cm^3^ min^−1^ (for calculating *J*_O_2__). Shell side: *F*_He_ = 40 cm^3^ min^−1^, *F*_CH_4__ = 10 cm^3^ min^−1^.

The oxygen permeation fluxes of coated/uncoated BCFZ hollow-fiber membrane were measured in a high temperature permeation cell (see Figure 1) using inert He or diluted methane as sweep gas. As shown in Figure 3A, the oxygen permeation fluxes increased with temperature raising for the coated and uncoated membranes. The increase of oxygen permeation flux should result from the faster surface exchange rate and the improved diffusion kinetics of oxygen ion in the bulk part of perovskite membrane with increasing operation temperature. Moreover, it was noticed from Figure 3A that the oxygen permeation fluxes of the coated BCFZ hollow-fiber membrane is slightly higher than that of uncoated membranes when using He as the sweep gas, and an oxygen permeation flux of ca. 4.7 cm^3^ cm^−2^ min^−1^ was obtained at 950°C. The oxygen permeation flux of BCFZ hollow-fiber membrane is limited by surface exchange rate and bulk diffusion rate of oxygen ions based on our previous study (Jiang et al., 2010b). After depositing a BCFZ porous layer on the outside surface of BCFZ hollow-fiber membrane, there are more active sites on the permeate side (shell side) for the association of oxygen ions to form molecular oxygen, which is followed by desorption. This results in a slightly higher surface exchange rate. Furthermore, when diluted methane (instead of inert He) was sent to the permeate side, the porous layer can well catalyze the combustion of methane (CH4 + 4 O^2−^ → CO_2_ + 2 H_2_O + 8e^−^). In this case, the permeated oxygen can be reacted quickly by combustion, leading to a larger gradient of oxygen partial pressure across the membrane, thus a higher oxygen permeation flux can be expected. Figure 3B presents the oxygen permeation fluxes of the uncoated and coated BCFZ hollow-fiber membranes at different temperatures feeding diluted methane on the permeate side. Compared with the uncoated BCFZ membrane, oxygen permeation flux increased from 4.6 to 15.9 cm^3^ min^−1^ cm^−2^ at 950°C after being coated with a porous BCFZ layer.

**Figure 4:**
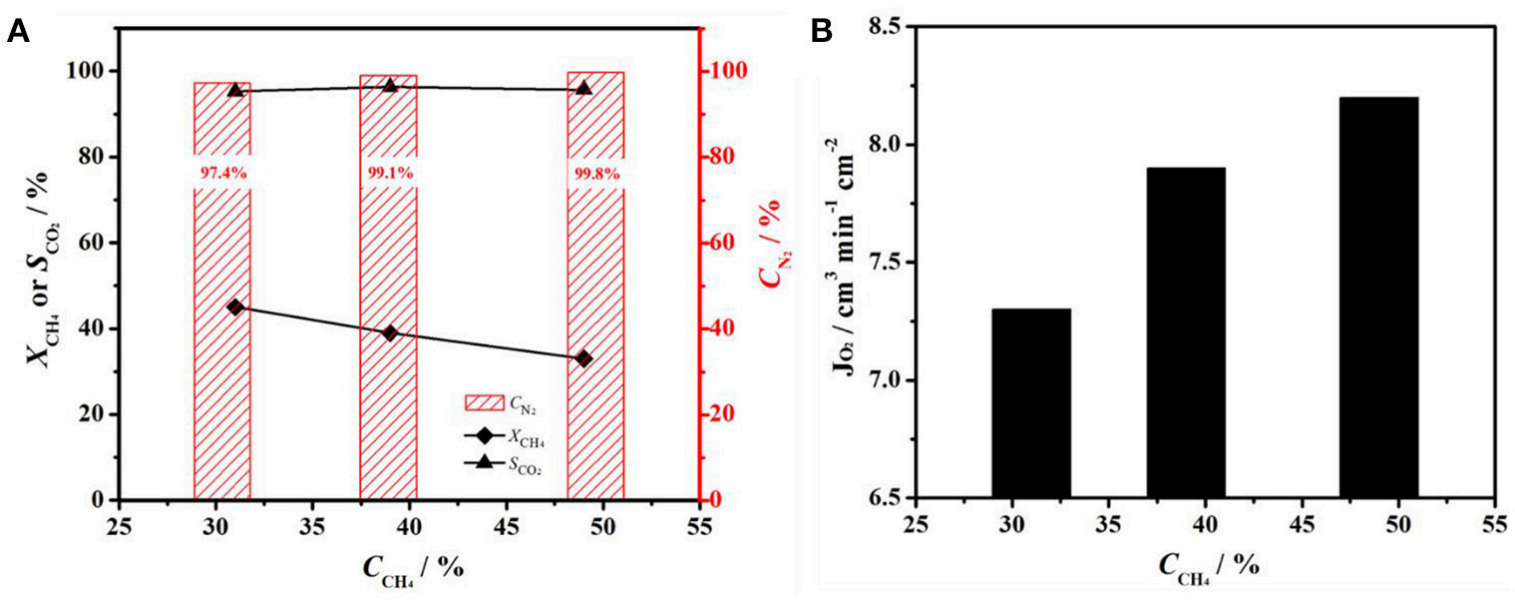
**(A)** Nitrogen purity (*C*_N_2__), CH_4_ conversion (*X*_CH_4__) and CO_2_ selectivity (*S*_CO_2__), and **(B)** oxygen fluxes (*J*_O_2__) as a function of methane concentration on a BCFZ hollow-fiber membrane with porous layer at 875°C. Membrane area: 1.1 cm^2^. Core side: *F*_Air_ = 48 cm^3^ min^−1^. Shell side: *F*_CH_4__ + *F*_He_ = 30 cm^3^ min^−1^.

From the above results, it can be demonstrated that oxygen permeation fluxes are significantly enhanced by coating BCFZ hollow-fiber membrane with a porous BCFZ layer. Thus, such BCFZ hollow-fiber membrane with a porous catalytic layer was employed to produce nitrogen by optimizing the operation conditions. In BCFZ membrane reactor, oxygen molecules from air on core side are removed as oxygen ions (O^2−^) through membrane to shell side, where they are consumed quickly by methane combustion, as illustrated in Figure 1B. During this process, oxygen can be continuously removed from air, which leads to a gradual increase on the purity of nitrogen. Thus, methane concentration on shell side plays an important role on influencing nitrogen purity on core side. Figure 4 shows the effect of methane concentration on the performance of the coated BCFZ membrane reactor. With methane concentration on shell side increasing from 31 to 49%, it was found that the nitrogen purity of outlet gas on core side increased from 97.4 to 99.8% according to Figure 4A. This purity meets requirements of many application fields in chemical industry, such as shielding gas or purging gas. Attributed to methane concentration increasing, oxygen on shell side would be consumed faster. Thus, the residual oxygen partial pressure on the shell side would decrease, which leads to a higher oxygen permeation flux. In our work, oxygen permeation flux increased from 7.3 to 8.2 cm^3^ min^−1^ cm^−2^ as methane concentration increasing from 31 to 49%, as shown in Figure 4B. Therefore, higher nitrogen purity was achieved with increasing methane concentration on the permeate side. Accordingly, the methane conversion decreased significantly from 45 to 33% upon increasing methane concentration from 31 to 49% (Figure 4A). Compared with the increment of methane, the oxygen supply was not enough for the total conversion of methane although oxygen permeation rate increased from 7.3 to 8.2 cm^3^ min^−1^ cm^−2^, leading to a lower methane conversion. It is noticed that the carbon dioxide selectivity remained 95% in all cases, which indicated the Co-based BCFZ oxides prefer to catalyze the total oxidation of methane rather than the partial oxidation to CO.

**Figure 5:**
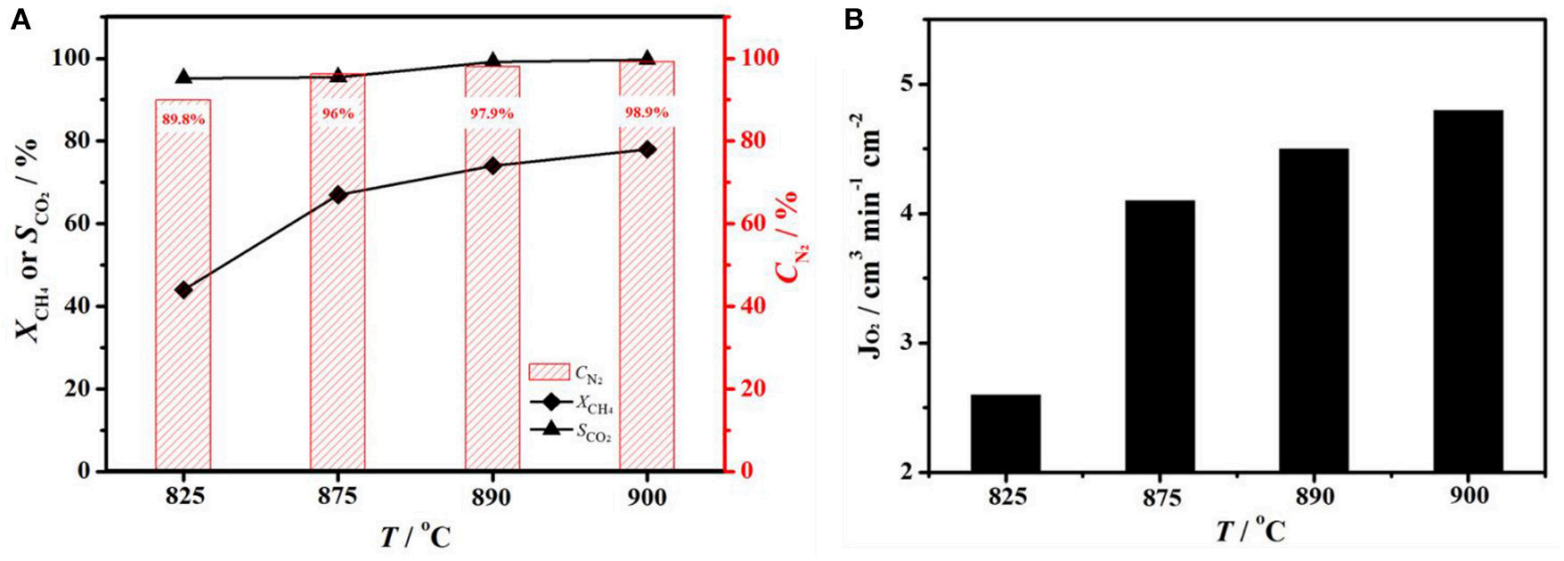
**(A)** Nitrogen purity (*C*_N_2__), CH_4_ conversion (*X*_CH_4__) and CO_2_ selectivity (*S*_CO_2__), **(B)** oxygen fluxes (*J*_O_2__) as a function of temperature on a BCFZ hollow-fiber membrane with porous layer. Membrane area: 1.1 cm^2^. Core side: *F*_Air_ = 29 cm^3^ min^−1^. Shell side: *F*_CH_4__ = 3.5 cm^3^ min^−1^, *F*_He_ = 7 cm^3^ min^−1^.

In order to achieve nitrogen production with a higher methane conversion on the other side of membrane, one can increase the operation temperatures. The influence of operation temperature on performance of BCFZ membrane reactor was shown in Figure 5. When temperature was 825°C, nitrogen purity and methane conversion was 89.8 and 44%, respectively (Figure 5A). With temperature raising, both nitrogen purity and methane conversion increased significantly. When temperature reached 900°C, nitrogen purity of 98.9% and methane conversion of 78% was obtained. Based on Wagner equation (Jiang et al., 2008; Chen et al., 2014), oxygen permeation rate is promoted by increasing operation temperature. Thus, more oxygen (*J*_O_2__ increased from 2.6 to 4.8 cm^3^ min^−1^ cm^−2^, as shown in Figure 5B) was removed from core side to shell side and consumed by methane oxidation there. Owing to the higher oxygen permeation flux, both nitrogen purity on core side and methane conversion on shell side increased, and carbon dioxide selectivity almost kept constant.

**Figure 6:**
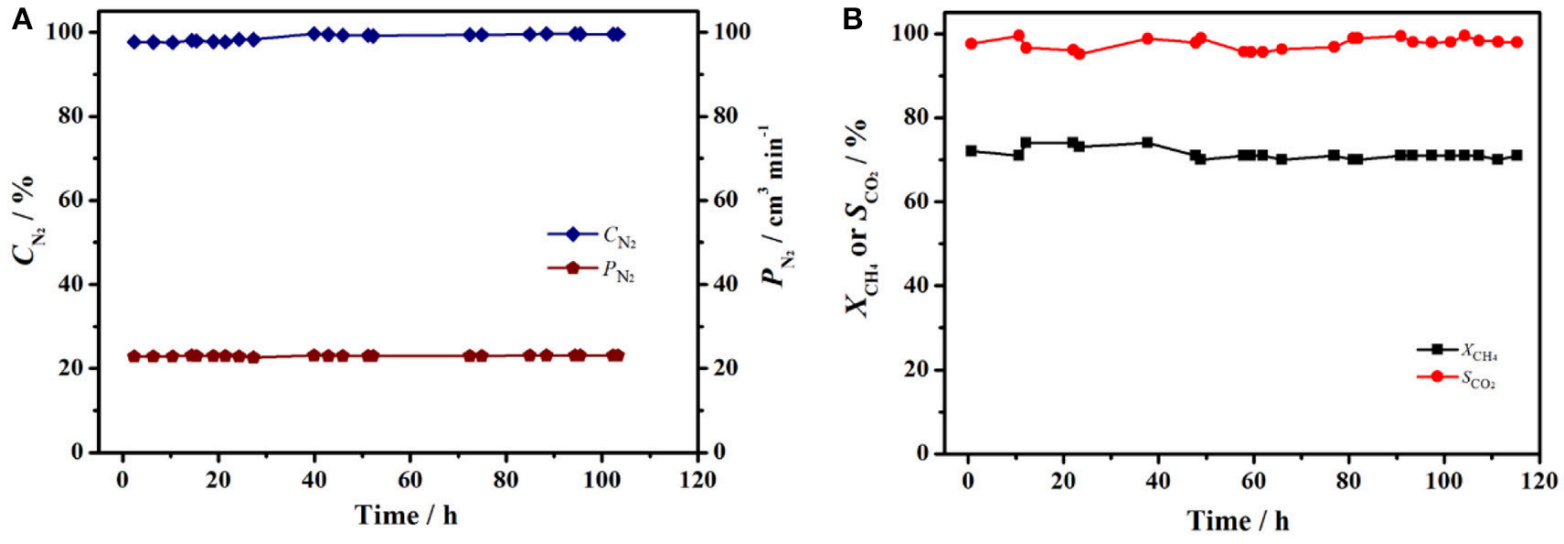
Long time stability test of BCFZ hollow-fiber membrane with porous layer at 890°C. **(A)** Production rate of nitrogen (*P*_N_2__) and nitrogen purity (*C*_N_2__),**(B)** CH_4_ conversion (*X*_CH_4__) and CO_2_ selectivity (*S*_CO_2__). Membrane area: 1.1 cm^2^. Core side: *F*_Air_ = 29 cm^3^ min^−1^. Shell side: *F*_CH_4__ = 3.5 cm^3^ min^−1^, *F*_N_2__ = 7 cm^3^ min^−1^.

**Figure 7:**
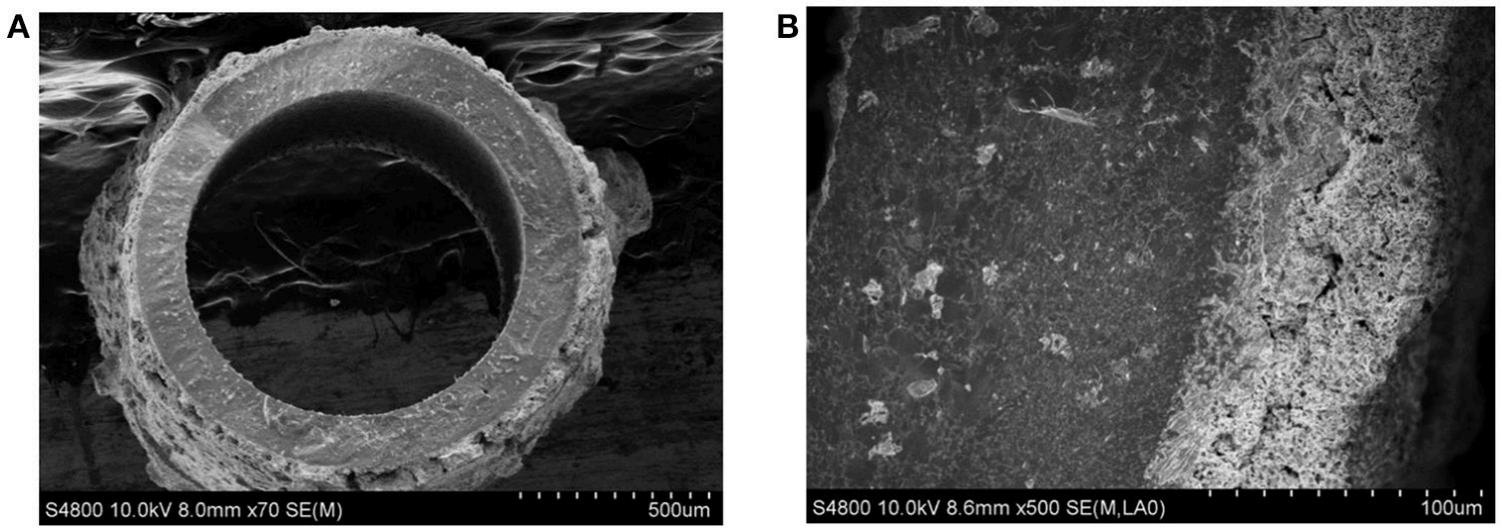
The SEM images of the BCFZ hollow-fiber membrane with porous layer after 120 h operation. **(A)** Cross-section micrograph and **(B)** magnified one.

The stable operation to produce nitrogen from air using perovskite BCFZ hollow-fiber membrane with a porous catalytic layer was conducted. During the test period of 120 h, nitrogen was produced at a rate of ca. 23 cm^3^ min^−1^ with a purity of 98–99% on core side (Figure 6A), and a methane conversion of 71–73% with a carbon dioxide selectivity of above 95% were also achieved (Figure 6B) on shell side at 890°C. The nitrogen production from air using ceramic catalytic membrane driven by methane oxidation seems promising based on our study. In addition, as shown in Figure 7A, the porous layer is still well coated on outside surface of BCFZ hollow-fiber membrane after 120 h operation, and according to magnified image (Figure 7B), the surface layer is still porous, which supports the stable performance.

## Conclusions

In summary, we demonstrated the nitrogen production using a perovskite oxygen-permeable membrane with a porous catalytic layer, in which air separation was coupled with methane combustion on the opposite sides of membrane reactor. Based on *in-situ* and fast removal of oxygen in air by using the oxygen-permeable membrane, nitrogen was produced at a rate of ca. 23 cm^3^ min^−1^ with a purity of 98–99% on core side of BCFZ hollow-fiber membrane. On shell side, a methane conversion of 71–73% and a carbon dioxide selectivity of above 95% were also achieved at 890°C and it can be steadily operated for over 120 h. The joint use of oxygen-permeable membrane and methane combustion seems a promising and efficient strategy for nitrogen production. This technology seems economically attractive especially when the low quality of methane such as the flare gas in the offshore platform is used to drive the membrane-based separation. This will also inspire more research efforts in the field of gas separation using perovskite catalytic membrane.

## Author contributions

HP and HJ conceived and designed the experiments; TH and HZ conducted the experiments and analyzed the data; TH wrote the draft, and HP and HJ improved it.

### Conflict of interest statement

The authors declare that the research was conducted in the absence of any commercial or financial relationships that could be construed as a potential conflict of interest. The reviewer SW and handling Editor declared their shared affiliation.
